# Association between different dietary patterns and eating disorders and periodontal diseases

**DOI:** 10.3389/froh.2023.1152031

**Published:** 2023-03-22

**Authors:** Maísa Casarin, Taciane Menezes da Silveira, Beatriz Bezerra, Flavia Q. Pirih, Natália Marcumini Pola

**Affiliations:** ^1^ School of Dentistry, Federal University of Pelotas, Pelotas, Brazil; ^2^ School of Dentistry, Section of Periodontics, University of California, Los Angeles, CA, United States

**Keywords:** inflammation, diet, nutrition status, periodontitis, feeding and eating disorders

## Abstract

Periodontal diseases is a highly prevalent chronic condition regulated by the host immune response to pathogenic bacterial colonization on the teeth surfaces. Nutrition is a critical component in the modulation of the immune system, hence the importance of a balanced diet. With the understanding of how dietary intake composition affects various health outcomes, nutrient diversity has been reported as a modifiable risk factor for periodontal disease. Eating disorders and different dietary patterns can be associated with periodontal diseases. In this sense, balanced and healthy nutrition plays a major role in maintaining the symbiosis between oral microbiota and periodontal health. Therefore, this review seeks to report the associations found in the literature between high- or low-fat/sodium/sugar, eating disorders and periodontal diseases. It was found that some dietary patterns such as high carbohydrate/sugar, high fat, and low fiber intake may be associated with periodontal disease. In addition, the presence of eating disorders can negatively impact patients’ oral health and it is related to the development of several complications, including periodontal diseases. In both situations, nutritional and vitamin deficiencies can aggravate the periodontal condition. However, the relationship between periodontal disease, dietary patterns, and eating disorders still needs more scientific support to be well established, mainly in the sense of pointing out a protective relationship between both.

## Introduction

Nutrition is essential for the lifelong development of human beings (1). Better nutrition improves health, reinforces the immune system, contributes to longevity, and decreases the chance of non-communicable diseases (2). Thus, nutrition and health are strongly connected. Currently, there is a double burden of malnutrition worldwide, including undernutrition and overnutrition. Both forms of dietary deficiency are a major challenge to human health (3). According to the Global Nutrition Report published in 2020, one in nine people worldwide experiences hunger or is undernourished, and one in three is overweight or obese (4).

**Figure 1:**
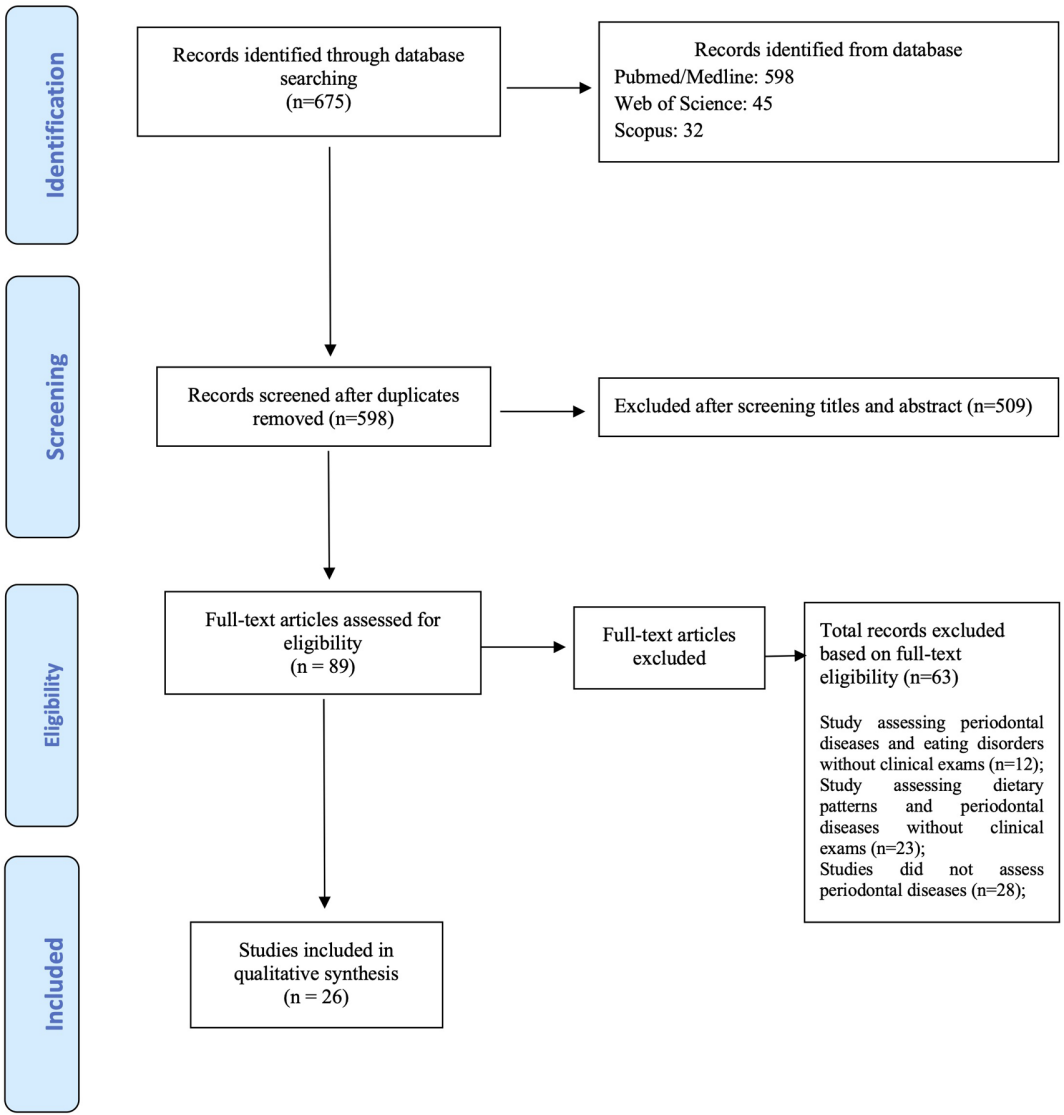
Flowchart.

Nutrition and oral health are connected, and dietary patterns play primarily a modifying factor in the progression of periodontal disease (5). Periodontal diseases are among the most prevalent human inflammatory diseases. The international age-standardized prevalence and occurrence of new cases have remained stable since 1990 when the prevalence was at 11.2% and incidence at 696 cases per 100,000 person-years. Compared to a prevalence of 10.8% and an incidence of 701 cases per 100,000 person-years in 2010 (6). Periodontal disease is an inflammatory process that occurs in the periodontal tissues in response to bacterial biofilm accumulation in the periodontium. In susceptible individuals, periodontal disease can cause destruction of collagen fibers in the periodontal ligament, and alveolar bone resorption, leading to mobility or tooth loss (7). It is estimated that nearly 732 million people all around the world share common risk factors of many chronic diseases that contribute significantly to the global burden of oral diseases (8).

The evidence shows that a diet high in saturated fats and sucrose increases the risk of several chronic conditions such as cardiovascular diseases, diabetes, and cancers, with a diet rich in saturated fats and sucrose in comparison to a diet rich in low-energy foods, such as fruits and vegetables (9). Meanwhile, studies have suggested that maintenance of normal body weight through a healthy diet and physical exercise practice significantly reduces the prevalence of gingival inflammation and the severity of periodontitis (10, 11).

It is shown that the antioxidant effects of vitamins in a healthy diet, may have a positive impact on the prevention and treatment of periodontal diseases (1). Also, calorie restriction, with different dietary patterns or eating disorders, can decrease the expression of inflammatory cytokines (interleukin-6, interleukin-10, interleukin-12, tumor necrosis factor-alpha, interferon-gamma, and polymeric immunoglobulin receptor mRNA), and increase the expression of immuno-suppressive mediators (transforming growth factor-beta) (12). Thus, the aim of this literature review is to report the associations between different patterns, eating disorders and periodontal diseases.

### Methodological aspects

This study consisted in a literature review to evaluate the association of individuals with different dietary patterns and eating disorders with periodontal disease. The searches were performed by two independent reviewers in the main international databases (PubMed, Web of Science, and Scopus), in addition to a manual search. The search strategy used in all databases included the descriptors and MeSH (medical subject headings) terms: “(periodontal diseases) AND (feeding and eating disorders OR anorexia OR bulimia) AND (High-Fat Diet OR Reducing Diet OR Weight Reduction Diet OR Weight Loss Diet OR Dietary Restriction)”, without study design distinction. The articles were analyzed without restriction of year and language. Initially 675 potentially relevant records were identified from the search strategy for both search (dietary patterns and eating disorders). After examining the title and abstract, 509 studies were excluded because they did not meet the selection criteria. Of the 89 studies retained for a detailed review, only 26 studies fulfilled all of the selection criteria and were included in the qualitative analysis (Figure 1).

### Association between different dietary patterns and periodontal diseases

A dietary pattern is defined as quantity, variety, or combination of different foods and drinks. The intake of foods with different nutritional value, such as the consumption of high proportions of vegetable, animal fat, industrialized foods rich in sodium and sugar, are related to different dietary patterns (1, 13). The relationship between the source and quantity of calories ingested in different dietary patterns has been linked, at the oral and systemic level, to the development of chronic diseases, such as periodontal diseases.

Periodontal diseases are triggered by an inflammatory response to the resident oral bacteria, leading to the compromising of structures that surround and support the teeth (14). As balanced nutrition has a relevant role in maintaining the symbiosis between oral microbiota and periodontal health (15), nutrition and oral health are strongly connected.

When analyzing the intake of nutrients in the dietary patterns of 3,043 individuals, Hamasaki et al. (2017) observed that individuals in the group with the highest Community Periodontal Index—(CPI scores 3–4), consumed more carbohydrates (*p* = 0.039) (16). Of these, a higher percentage of calories was obtained from carbohydrates (*p* < 0.001) and had higher levels of copper (*p* = 0.026), vitamin B12 (*p* = 0.018) and folic acid (*p* = 0.011), but lower levels of vitamin C (*p* = 0.004), compared to individuals in the group with lowest CPI score 0–2. On the other hand, they consumed significantly less total fat (*p* < 0.001), which suggest that a low-fat, high-carbohydrate dietary pattern is related to periodontal disease (16).

Kondo et al. (2014) performed an interventional study assessing the effects of a high-fiber, low-fat diet on clinical parameters of periodontal disease in twenty-one volunteers. The volunteers had body mass index of at least 25.0 kg/m^2^ or intolerance to glucose (17). During the period of 8 weeks, the subjects that had a high-fiber, low-fat diet showed effectively improvement in probing pocket depth (PD), clinical attachment level (CAL), and bleeding on probing, as well as metabolic profiles, at least in part through effects other than reduced fat intake (17).

Data from the British Regional Heart Study (BRHS) in the United Kingdom and Health, Aging, and Body Composition in the United States show poor oral health in older adults is associated with poor diet quality, higher total energy intake and percentage of the energy content of saturated and trans-fat, low intake of fruits and vegetables, and high intake of processed meat (18). And in the National Health and Nutrition Survey (NHANES III) data cohort, high intake of added sugar was associated with a higher PD prevalence ratio of 1.42 (95% CI, 1.08–1.85) (19).

A decrease in the risk of gingival infection is also reported when carbohydrate consumption is restricted on a four-week diet (10). The presence of high rates of sugar or refined carbohydrates promoted microbiota dysbiosis, which can induce an inflammatory response, leading to the presence of deeper periodontal pockets (20). Furthermore, it is demonstrated that glucose acts on periodontal ligament cells, promoting apoptosis and inhibiting cell proliferation (21). Apart from sugar restriction, one study showed that sugar substitutes are not fermented by the microflora of the dental plaque (22). The literature reports that xylitol, produced by the hydrogenation of xylose sugar, showed antibacterial effect against *Porphyromonas gingivalis* and *Aggregatibacter actinomycetemcomitans,* both periodontopathogenic bacterias (23).

A prospective cohort study of 6,209 participants conducted at the University Medical Center Hamburg-Eppendorf assessed subjects went on a dietary approach considered to stop hypertension (DASH) (plant-based foods, low-fat dairy, fish, and whole grains) and the Mediterranean diet (rich in plant-based foods, fish, and olive oil, based on low consumption of red meat and processed foods) (9). The results of the study showed a significant association between participants’ adherence to the dietary patterns and a decreased probability of being diagnosed with periodontal diseases (OR: 0.92; 95% CI: 0.87, 0.97; *p* < 0.001 and OR: 0.93; 95% CI: 0.91, 0.96; *p* < 0.001, respectively) (9).

A randomized clinical trial with participants aged 65 to 75 years reported the consequence of protein sources on periodontal health, with a BMI between 20 and 35 kg/m^2^ (14). They had *ad libitum* access to one of four experimental diets (high-fat or high-carbohydrate omnivore, high-fat or high-carbohydrate semi-vegetarian) for 4 weeks. Between baseline and follow-up, the number of sites with CAL 1 mm and CAL gain > 1 mm calculated significant differences between the investigated diets. The authors concluded that a high-fat, semi-vegetarian diet has clinically relevant positive effects on clinical parameters of periodontitis, including PD and CAL reduction.

The effects of caloric restriction and intermittent fasting on periodontal health has also been studied (12). An improvement and extension of life expectancy has been reported in several animal models involving intermittent fasting or continuous restriction of caloric energy intake, without causing malnutrition (12).

In an experimental disease model, caloric restriction decreased gingival inflammation (*p* < 0.0001) and periodontal degradation (PD *p* < 0.0016; and CAL *p* < 0.0038) in male and female primates equally, when compared to control groups. These outcomes corroborate with the hypothesis that caloric restriction modulates the periodontal degradation by decreasing the inflammatory response (24). In a recent investigation conducted in an experimental model of periodontitis in rats, the beneficial effects of fasting regimens on periodontal tissues were evaluated. Peripheral quantitative computed tomography and calcein-labeled histomorphometric analyzes presented less amount of bone loss in the fasting group than in the non-fasting group (*p* < 0.05) (25). And femoral bone marrow cells from fasted groups produced more mineralized modules than non-fasted groups (25).

### Association between eating disorders and periodontal diseases

Eating disorders (EDs) are serious conditions defined as eating-related behaviors, considered psychiatric disorders (26, 27). These disorders result in inappropriate consumption or absorption of food that significantly decreases physical health or psychosocial functioning, the most frequent being Anorexia Nervosa (AN), Bulimia Nervosa (BN) and binge-eating disorder (28). AN is described by malnutrition, leading to a significantly low body weight, and food restriction. BN is characterized by binge eating, with a lack of control during these episodes, followed by inappropriate compensatory behaviors such as self-induced vomiting, use of laxatives to prevent weight gain, and over-exercising (29). Binge-eating disorder is characterized by recurrent episodes of binge eating, during which the individuals consume a large amount of food, but without the inappropriate compensatory behaviors from BN.

Oral health complications are usual features in patients with EDs, including dry lips, labial erythema, exfoliative cheilitis, palatal tissue discoloration, hemorrhagic lesions, burning tongue, dental erosion, swelling of the parotid glands, tooth decay and periodontal diseases (30–33). The oral complications are mostly caused by nutritional deficiencies and consequent metabolic impairment. Besides, it should be noted that, as these eating disorders have serious psychiatric comorbidities, these may be associated with poor oral hygiene, which can help in the development of periodontal disease (32).

Two case-control studies from Australia, with the same sample population, showed that 15 female patients with AN had more sites with bleeding on probing and gingival recession than controls (34, 35). Another case-control study with 33 women, 18 with AN and 15 with BN, and 33 age-matched women without history of eating disorder did not show differences on bleeding on probing. However 56.25% of the patients with eating disorder had periodontitis (defined as gingival recession or PD >3 mm) compared to 6% of the control group (36). Another cohort of 23 adolescents and young woman with restricting AN showed that 17.4% of the subjects had a simplified oral hygiene index score of ≥1, and 43% of the patients had gingival recession of at least 1 mm on ≥3 dental sites (37). Another cohort study including 7 patients with AN and 10 with BN showed that gingival bleeding was present in 28.6% and 30.0% of patients with AN and BN respectively; gingival recessions were noted in 14.3% of patients with AN and 40% with BN (38).

One cohort study analyzed only plaque index in 40 adults (30 woman and 10 men) with eating disorder and found that 45% of these patients exhibited an interproximal plaque index >70% (39). A recent case-control study in France with 70 women, 36 with AN and 34 with BN, and 70 women matched by age, noted a mean plaque index of 78.8% for AN and 63.7% for BN compared to 53.0% for controls. Mean bleeding on probing was 41.3% in AN subject, 18.5% in BN subjects, whereas control subjects presented 21.8%. Furthermore, gingival recession >2 mm was present in 2.3% of subjects with eating disorders compared with 0.0% in controls; the percentage of sites with clinical attachment level >2 mm was 33.9% in AN subjects compared to 22.9% in BN. However, when assessing the percentage of sites with PD >3 mm, only 0.5% of patients with eating disorder showed this finding when compared to 3.1% of controls (40).

A secondary analysis of the same case-control study reported above (40) showed data from 45 women, 18 with BN and 27 with AN. In this sample 48.1% of AN subjects and 28.5% of BN subjects presented ≥30% of sites with clinical attachment level ≥3 mm (41). Another case-control study with 54 patients (50 females and 4 males), 14 with AN, 8 with BN and 32 with unspecified eating disorders, compared with sex and age-matched controls, found different results with a median of gingival bleeding index of 1.0% in eating disorder compared to 7.1% in control group (42). A recent cross-sectional study analyzed 30 patients with an eating disorder and compared with 30 patients without an eating disorder. Differences were observed according to mean of community periodontal index between the presence of calculus of 1.60 for the eating disorder group compared to 0.10 for control group; and between bleeding on probing of 1.87 compared to 0.33 for the control group. Periodontal health was found to be 5.53 for the control group compared to 2.07 for the eating disorder. However, for periodontal pocket of 4–5 mm, no differences were found between groups (43).

## Discussion

Some dietary patterns such as high-carbohydrate/sugar, high-fat, and low-fiber intake can be associated with periodontal diseases. Besides, eating disorders can deteriorate patients’ oral health and are related to the emergence of several complications, including periodontal diseases. In both conditions, nutritional and vitamin deficiency can aggravate periodontal diseases.

Even though more scientific support is still necessary, the relationship between periodontal diseases and dietary patterns points in a clear direction, that a balanced dietary pattern rich in nutrients and vitamins can act as a protective factor for periodontal disease (1, 9, 12, 18). The function of micronutrients, such as vitamins D, E, K, and magnesium, is still uncertain, while others, such as vitamins A, B, C, calcium, zinc, and polyphenols have demonstrated potential to prevent PDs (1). These data refer to both the population level and the individual approach, considering that a healthy dietary pattern can prevent other chronic disorders (1, 11, 16, 18).

Most of the data presented for dietary pattern come from cross-sectional studies, which makes a cause-and-effect relationship impossible. The survey sources have limited data specificity, such as the types of fat and carbohydrate consumed, in addition, the periodontal indices used in large samples may underestimate the prevalence of the periodontal disease. Also, one should be paid attention to the different results found in controlled trials (10, 11, 14), which demonstrates the need for more investigations in the area.

Although there are still conflicting results in the literature about the association between eating disorders, dental plaque and gingival inflammation, there is a tendency for higher rates of plaque and gingival inflammation in individuals with eating disorders (34–36, 40, 42, 43). Regarding the occurrence of PD ≥3 mm, the available studies identified no differences between patients with eating disorders and controls groups (40, 43). However, the gingival recession in eating disorder was a consistent finding in the studies (38, 40). This finding can be associated with the maintenance of oral hygiene in patients with AN and BN. Studies suggest that patients with eating disorders can show less interest in maintaining oral health hygiene because of their depressive condition (44), whereas other patients may present a high frequency of toothbrushing practice (38, 40, 45, 46).

The several methods used in data collection of periodontal variables used in the studies may have influenced the results and associations. The use of different criteria to assess gingival inflammation (36–38, 40–43), dental plaque (39, 40, 42), probing pocket depth (36, 37, 40, 41, 43), clinical attachment level (41), community periodontal index (34, 35, 43), gingival recession (34, 35, 36, 38, 40) or other tools, as well as partial-mouth periodontal examinations (34, 35, 36, 39, 43), can underestimate the prevalence or, in some cases, overestimate the gradation of periodontal diseases (47–49). As well as the different definition of periodontal diseases, as Community Periodontal Index (16), probing depth (9, 14, 17, 24), clinical attachment loss (9, 14, 17–19, 24), bleeding on probing (9, 14, 17, 24), plaque index (24), gingival index (24), and bone loss (25), can interfere with the association between nutrition patterns and eating disorders. A number of comprehensive psychiatric interviews can be used to diagnose eating disorders, as a physical examination (50) and others. Different criteria for dietary patterns, as well as, for diagnosis and classification of eating disorders can affect the association between the condition and periodontal diseases. Nonetheless, many studies use self-report/questionnaire as a dietary assessment method, which may underestimate the consumption of foods socially considered unhealthy (9, 18). These contrasts emphasize the complexness of the relationship between periodontal disease and dietary patterns and eating disorders.

Oral health professionals may be the health professional most likely to suspect of an eating disorder or different dietary patterns due to oral effects of inappropriate diet, psychotropic medication, and self-induced vomiting. In this sense, there is a need to better quality oral health professionals for wide care of the patient with eating disorders and distinct dietary patterns. The oral health professionals must be part of a multidisciplinary team that includes psychiatrists, psychologists, nutritionists, and physicians in order to better assist these patients and help manage current and future oral health issues that include but are not limited to periodontal diseases.

## Conclusion and future perspectives

It is apparent from the available evidence that a nutritionally unbalanced dietary pattern high in fat, sodium and sugar, or the presence of eating disorders affects periodontal health. However, the association between periodontal disease and dietary quality and eating disorders still needs more scientific support to be well established, especially to point a protective relation between both. The oral health professionals should be aware of the oral health signs related to an eating disorder or dietary patterns.
